# Integrated coplanar waveguide coil on diamond for enhanced homogeneous broadband NV magnetometry

**DOI:** 10.12688/openreseurope.16875.1

**Published:** 2024-03-01

**Authors:** Hossein Babashah, Elena Losero, Christophe Galland

**Affiliations:** 1 Institute of Physics, Ecole Polytechnique Federale de Lausanne, Lausanne, Vaud, Switzerland; 2 Wainvam-e, Lorient, France; 3 Center for Quantum Science and Engineering, Ecole Polytechnique Federale de Lausanne, Lausanne, Vaud, Switzerland; 4 Division of Quantum Metrology and Nanotechnologies, Istituto Nazionale di Ricerca Metrologica, Torino, 10135, Italy

**Keywords:** Nitrogen-vacancy centers, Diamond quantum sensors, Magnetometry, Coplanar planar waveguide coil, Microwave field homogeneity, Broadband antenna design, Diamond substrate integration.

## Abstract

Nitrogen-vacancy (NV) centers in diamond have emerged as promising quantum sensors due to their highly coherent and optically addressable spin states with potential applications in high-sensitivity magnetometry. Homogeneously addressing large ensembles of NV centers offers clear benefit in terms of sensing precision as well as in fundamental studies of collective effects. Such experiments require a spatially uniform, intense, and broadband microwave field that can be difficult to generate. Previous approaches, such as copper wires, loop coils, and planar structures, have shown limitations in field homogeneity, bandwidth, and integration in compact devices. In this paper, we present a coplanar waveguide (CPW) gold coil patterned on a 3 × 3 mm ^2^ diamond substrate, offering full integration, enhanced stability, and broad bandwidth suitable for various NV sensing applications. Coil fabricated on diamond offers several advantages for magnetometry with NV centers ensemble, including enhanced heat dissipation, seamless integration, scalability, and miniaturization potential. We optimize critical geometrical parameters to achieve a homogeneous magnetic field with a coefficient of variation of less than 6% over an area of 0.5 mm ^2^ and present experimental results confirming the performance of the proposed CPW coil.

## Plain language summary

In recent years, there has been significant interest in using nitrogen-vacancy (NV) centers in diamond as quantum sensors for high-sensitivity magnetometry. These NV centers, particularly the negatively charged ones, offer promising applications due to their coherent spin states that can be manipulated using microwave fields and optically detected magnetic resonance techniques. However, to improve measurement precision and signal-to-noise ratio, it’s advantageous to address large ensembles of NV centers, which requires a spatially uniform, intense, and broadband microwave field. Various methods, such as copper wires, loop coils, and planar structures, have been explored to achieve this, but with limited capability. To address their limitations, a coplanar waveguide (CPW) gold coil patterned on a CVD diamond substrate is proposed. This design offers a highly homogeneous magnetic field, full integration with the diamond substrate, scalability, miniaturization, and efficient heat dissipation, making it a promising solution for NV magnetometry applications. Experimental results confirm its performance, making it a remarkable advancement in this area.

## 1 Introduction

The investigation of nitrogen-vacancy (NV) centers in diamond as quantum sensors has attracted considerable interest in recent years, driven by their promising applications in high-sensitivity magnetometry ^1– 3^. Specifically, the negatively charged NV center has been the subject of extensive research due to its highly coherent spin states, which can be controlled through microwave fields and polarised and probed using continuous wave optically detected magnetic resonance (CW-ODMR) techniques ^4^.

Whenever nanoscale spatial resolution is not targeted, it is advantageous to address large ensembles of NV centers in order to improve the signal-to-noise ratio and enhance the measurement precision, theoretically scaling as $1/\sqrt{N}$ for *N* identical color centers contributing to the signal. This advantage is only fully leveraged under a spatially uniform, intense, and broadband microwave field, which should ideally be generated within a compact and integrated design ^5^. Achieving these conditions ensures optimal performance and compatibility across various applications, spanning the fields of medical imaging, nanoscale nuclear magnetic resonance (NMR) spectroscopy ^6^, quantum information processing ^7^, geophysical exploration ^8^, navigation and positioning ^9^, magnetic microscopy ^10^, and high dynamic range magnetic field sensing ^11, 12^.

The primary challenge lies in generating highly homogeneous broadband microwave fields over a large area at the center frequency of 2.87 GHz. Various methods have been explored to overcome this challenge, with mixed results. Copper wires, for instance, are capable of generating intense magnetic fields, which are useful for single NV centers ^13^. However, they exhibit poor magnetic field homogeneity. Furthermore, the alignment of the copper wires with the NV centers can lead to substantial variations in microwave magnetic field strengths ( *B*_1_), affecting the performance of the magnetometer. Loop coils offer an alternative approach and produce a *B*_1_ field with superior homogeneity ^14^. However, this field is relatively low, limiting their effectiveness. Researchers have turned to planar structures on different dielectric substrates in an attempt to address these issues.

Bayat *et al*. ^15^ developed a double split ring resonator, which generates spatially uniform magnetic fields and efficiently couples to NV centers. While the resonator does produce a *B*_1_ of around 0.78 Gauss, it suffers from a low bandwidth of 40MHz. Sasaki ^16^ introduced a planar ring antenna that generates a higher *B*_1_ of 3.2 Gauss and has a larger bandwidth of 400 MHz. Despite these improvements, the design is not compact and it suffers from a poor reflection coefficient, which reduces the microwave excitation efficiency and requires the use of a microwave isolator to prevent potential damage to the driving circuit. Opaluch ^17^ proposed a planar antenna fabricated on a glass substrate, which presents a large bandwidth and a relatively high *B*_1_ of 2 Gauss. However, the homogeneous field area is limited to a maximum of 0.28 mm ^2^. Additionally, the design is not fully integrated on a typical diamond-sized substrate (3mm × 3mm) due to its large size, hampering the development of a fully integrated sensor. In summary, while each of these approaches offers certain advantages for NV- ensemble-based magnetometry, none provides an ideal solution. Further research and development are needed to create a planar antenna design that generates a spatially homogeneous, intense, and broadband microwave field while maintaining a compact, integrated form factor making it a good candidate for real applications.

To address these limitations, we propose a coplanar waveguide (CPW) gold coil patterned directly on the 3 × 3 mm ^2^ surface of a CVD diamond substrate, designed to achieve a highly homogeneous magnetic field over a large area of 0.5 mm ^2^. The proposed CPW coil offers the advantage of full integration with a diamond substrate hosting NV centers, which can significantly enhance the sensor’s stability and robustness. This design has the advantage of being both scalable and miniaturized, as well as providing efficient heat dissipation during the operation of the coil thanks to the diamond high thermal conductivity. Additionally, the CPW coil features a broad bandwidth suitable for sweeping microwave fields from 1.5 to 4 GHz, a requirement for certain NV applications ^16^. To facilitate ease of testing and operation, we also propose simple printed circuit board (PCB) and holder designs that eliminate the need for RF probe needles. Importantly, the magnetic field generated by the CPW is entirely perpendicular to the diamond plane. We have optimized critical geometrical parameters, such as the diameter of the omega-shaped resonator, the width of the waveguide, and the gap size, to achieve the desired magnetic field homogeneity. S-Parameter analysis and experimental results from Rabi measurements demonstrate good agreement with simulation results, confirming the performance of the proposed CPW coil. By addressing the challenges associated with earlier antenna designs, our CPW coil provides a more effective solution for NV magnetometry applications, combining homogeneity, broad bandwidth, and ease of use with the potential for full integration on a diamond substrate. The performance of the proposed antenna compared with the other options present in the literature is summarized in Table 1.

**Table 1.** Comparison of different antennas parameters.

Design	*B*_1_ (Gauss)	Field Area ( *mm* ^2^)	Bandwidth (MHz)	Dimension ( *cm* ^2^)
Bayat ^15^	0.78	4.5	40	2 × 2
Sasaki ^16^	3.2	3.1	400 ( *S*_11_ *<* –2 *dB*)	5 × 3
Oplauch ^17^	1.1	0.2	0-6200	1.6 × 1.1
This work	1.6	0.5	2000-2900	0.3 × 0.3

This paper is organized as follows: Section 2 discusses the antenna design and geometry, detailing the specific features that contribute to its performance. Section 3 delves into the fabrication process of the CPW on the diamond substrate, as well as the construction of the PCB stack. In Section 4, we analyze the antenna’s performance, comparing simulation and experimental results, and present the characterization of the NV ensemble using the proposed antenna. This assessment will demonstrate the effectiveness of the CPW coil in achieving the desired magnetic field and homogeneity. Finally, Section 5 provides a conclusion, summarizing the key advantages of the proposed CPW coil and its potential impact on the field of NV magnetometry.

## 2 CPW Design and Geometry

**Figure 1.**
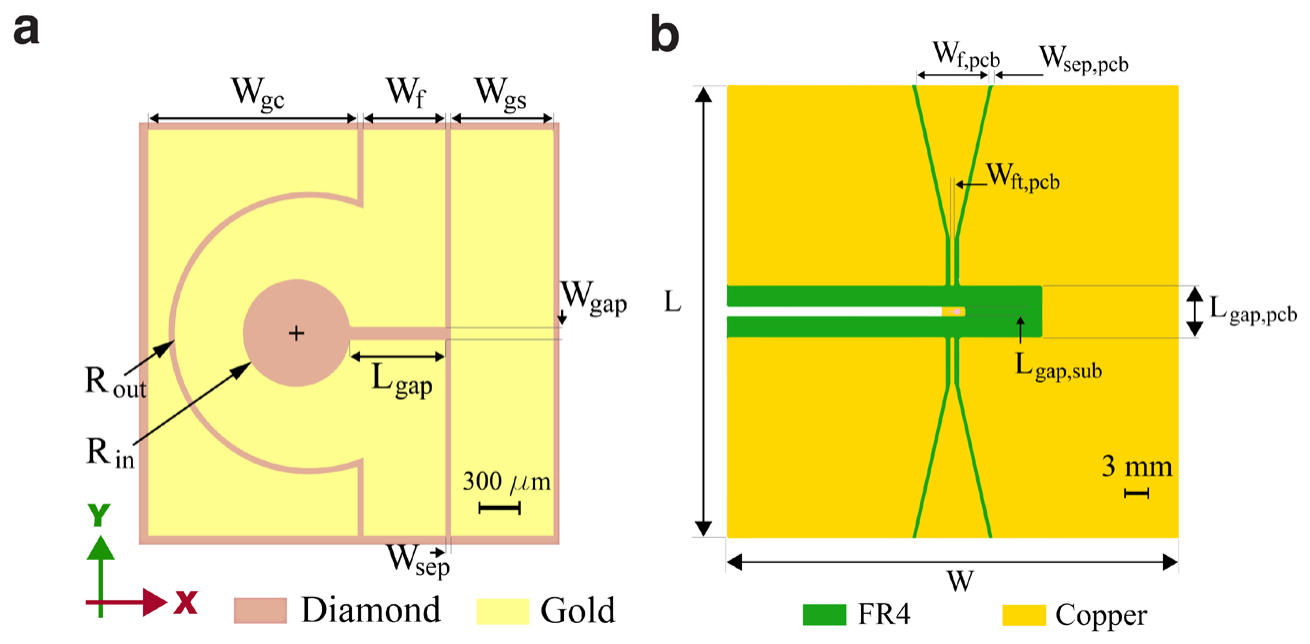
( **a**) Schematic diagram of the proposed antenna, patterned on a (3 × 3)mm ^2^ diamond chip and designed for efficient coupling of the microwave field to NV centers. ( **b**) Schematic diagram of the PCB holder for ease of excitation and characterization of the CPW antenna. The PCB design comprises two layers. On the top layer, a 50Ω coplanar waveguide with SMA connectors is patterned and truncated. The top layer is then connected to a transmission line in the bottom through three vias for the ground and excitation ports.

The design of the CPW coil has been performed through numerical simulations utilizing finite element method (FEM) techniques in ANSYS HFSS software. We began by parametrically defining a gold pattern, with all the parameters illustrated in Figure 1a. The gold pattern is simulated with a bulk conductivity of *σ* = 4.5 × 10 ^7^ S/m, the diamond substrates with dimensions 3.1 mm ×3.1 mm ×0.5 mm diamond substrate, featuring a thickness of 500 *μ*m and a relative permittivity of *ϵ _r_* = 5.7. The structure is excited through a 50Ω wave port. To avoid higher-order modes and ensure the correct fundamental mode is excited, we select a wave port size smaller than the lowest wavelength employed in the simulation. Additionally, we set the height of the wave port to be at least five times the thickness of the diamond substrate, and the width to be at least three times the width of the feed line denoted as *W _f_*. For the boundary conditions, we utilize a radiation box with dimensions ten times larger than the structure, which provides sufficient numerical stability throughout the simulation run time. Furthermore, a genetic algorithm optimization approach is employed to optimize the antenna parameters, including *W _f_*, *W _sep_*, *W _gap_*, and *L _gap_*. The optimization aims to maximize the *B*_1_ field generated at the coil’s center while minimizing the coefficient of variation (CV) for its magnitude at the green circles given in Figure 6a. Simultaneously, it enforces an acceptable reflection coefficient ( *S*_11_) of less than −10 dB. We set the convergence criterion, the maximum magnitude difference between successively calculated S-parameters, to be lower than 1 × 10 ^−5^ for each simulation.

In addition to the CPW design, a two-layer printed circuit board (PCB) and a 3D-printed holder has been designed to facilitate easy excitation and characterization of the antenna without necessitating an RF probe station setup. This configuration is achieved by placing the antenna between the PCB and the holder, which establishes the connection. We selected a PCB with a thickness of 800 *μ*m, composed of FR4 substrate with a relative permittivity of 4.4. Figure 1b depicts the schematic of the designed PCB, which employs a CPW design. The feed line width and separation are chosen to attain a 50 Ω impedance at the input and then truncated to gradually reach the CPW dimension limits. The CPW diamond coil is connected to the PCB inputs through three vias at each port. The optimized dimensions for both the diamond CPW coil and the PCB are provided in Table 2. Alternatively, the antenna can be excited without the need for a PCB by directly soldering micro-miniature RF connectors to the CPW on the diamond chip.

**Table 2.** Dimensions of the proposed CPW coil and printed circuit board (PCB), in *μ*m.

*W _gc_*	*W _f_*	*W _gs_*	*W _sep_*	*W _gap_*
1550	600	75	50	100
*L _gap_*	*R _in_*	*R _out_*	*W _f,pcb_*	*W _f t,pcb_*
700	400	950	8500	500
*W _sep,pcb_*	*L _gap,pcb_*	*L _gap,sub_*	*W*	*L*
500	6000	1000	53000	53000

## 3 Fabrication Process

**Figure 2.**
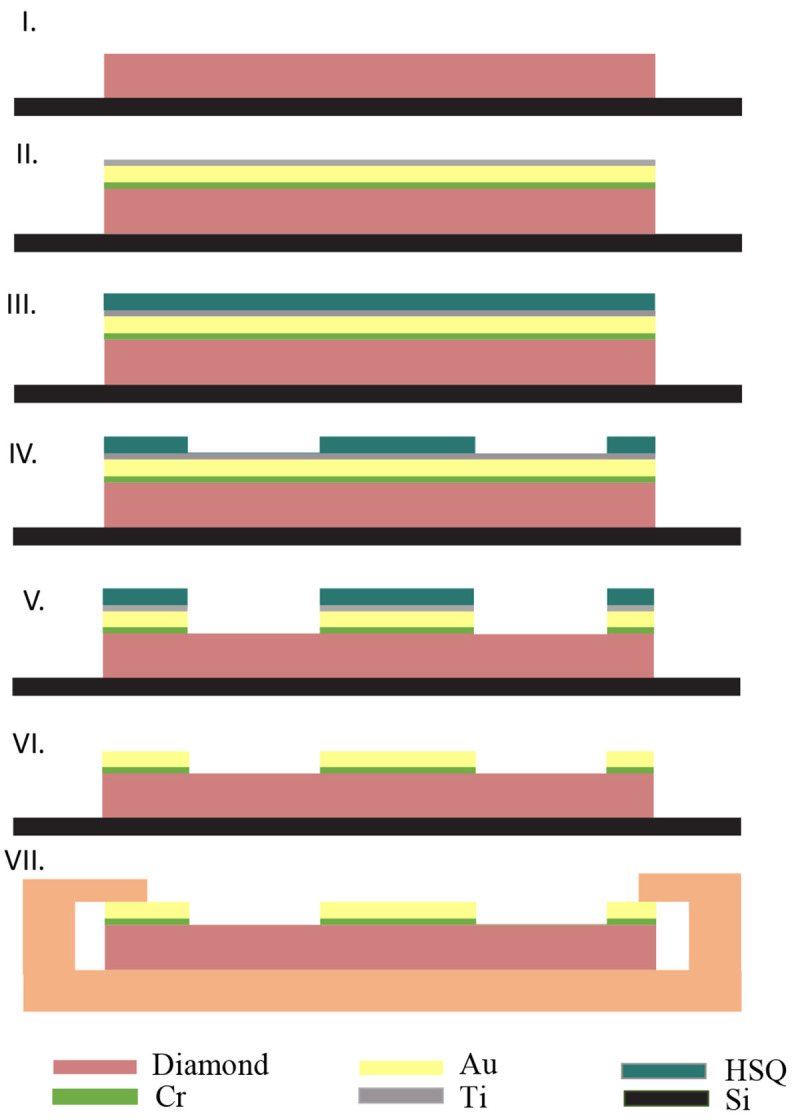
Fabrication process for our gold CPW antenna on diamond. **I**. Diamond substrate cleaning, non-contact fine polishing (see main text) and mounting on a Si carrier wafer. **II**. Evaporation of Cr (10nm) - Au (200nm) - Ti (10nm). **III**. Spin-coating of HSQ negative resist (150nm) **IV**. Electron beam lithography with the antenna design, and development in TMAH. **V**. Ion beam (Ar) etching of the Cr-Au-Ti multilayer. **VI**. Stripping of the HSQ and Ti layer in diluted hydrofluoric acid (HF). **VII**. Removal from the carrier wafer and possible integration in the PCB.

We fabricate our antenna on a commercially available 〈100〉 polished CVD-grown single crystal diamond substrate, presenting a uniform native NV concentration of ∼ 1.5 ppb (3.1 mm×3.1 mm×0.25 mm, optical grade, Element6). The fabrication process flow is depicted schematically in Figure 2. First, the sample is mounted using an adhesive (QuickStick mounting wax) on a standard Si wafer to facilitate handling and processing. To eliminate potential contamination, the sample is cleaned in acetone and then subjected to a piranha solution (3:1, H _2_SO _4_ : H _2_O _2_, 3 min) treatment.

In order to enhance the surface quality by removing residual mechanical polishing lines and pits and other surface defects, a preliminary non-contact polishing step is applied. This process employs the physical bombardment of the diamond surface with accelerated Ar gas ions (having an energy of 750 eV) at different incident angles. Further details of this process can be found in 18. Following our non-contact polishing, a root mean square (rms) roughness of less than 2 nm is measured over a 10 *μ*m ^2^ region. We then evaporate 10 nm of Cr, 200 nm of Au and 10 nm of Ti. Both Cr and Ti serve as adhesion layers. Afterward, we spin coat ∼ 150 nm Hydrogen silsesquioxane (HSQ XR-1541-006) negative resist, and pattern the antenna design using electron beam lithography. The critical dimensions of the antenna allow us to use a big pixel dimension and electron beam diameter, resulting in a fast exposure time of less than 2 minutes for the entire 3 mm × 3 mm chip. Subsequently, the development is done in Tetramethylammonium hydroxide (TMAH 25%) for 2 minutes. Alternatively, photolithography can be employed to significantly enhance the scalability of the patterning process.

We transfer the pattern from the resist to the Cr-Au-Ti layers using a collimated beam Ar ion etcher (Veeco Nexus IBE350, operation pressure 10 ^−4^ mbar, base pressure 10 ^−7^, Ar ^+^ ion energy 200 eV). At this energy, the metals are rapidly etched away (Au etching rate ∼ 50 nm/min), while the diamond is not damaged. Finally, the remaining HSQ and Ti layers are removed by diluted HF wet etch (1% concentration) and the antenna is removed from the carrier Si wafer.

**Figure 3.**
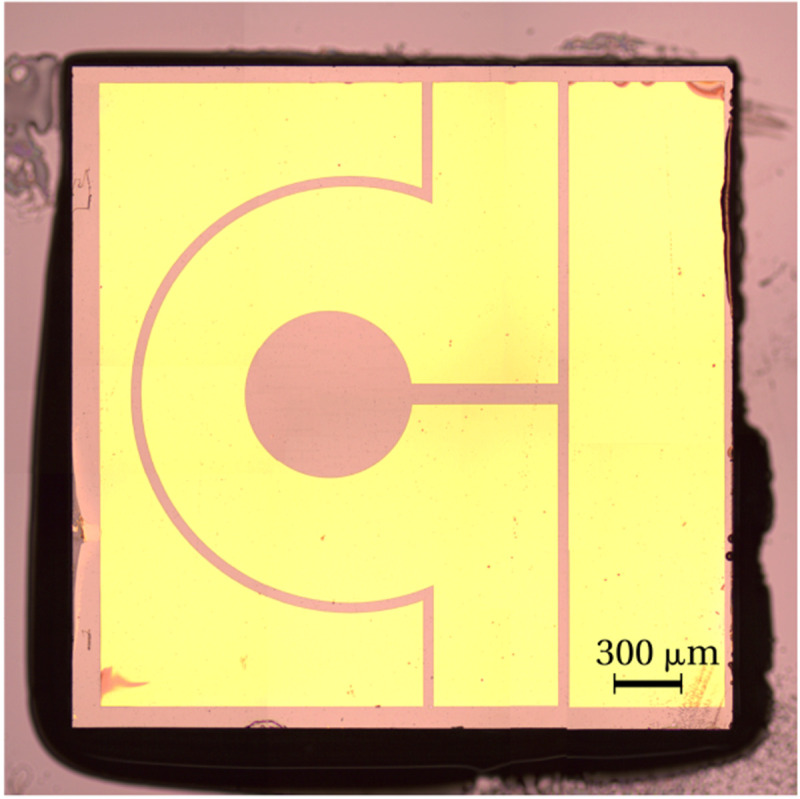
Optical image of the fabricated coplanar waveguide antenna fabricated on a diamond substrate.

**Figure 4.**
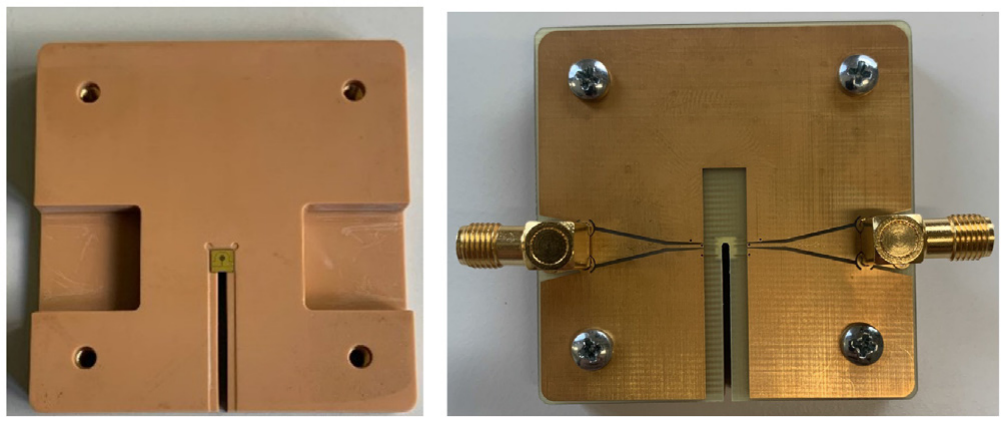
( **a**) 3D printed holder with the patterned diamond chip on top. The holder is designed to securely hold the CPW antenna and allow for easy integration with electronic circuits and optics. ( **b**) Assembled device: the diamond with the patterned CPW coil is sandwiched between the PCB and the 3D-printed holder.

The optical microscope image of the resulting antenna is shown in Figure 3. To prepare the antenna for testing, we mount it on the 3D printed holder ( Figure 4a). Then the antenna is sandwiched between the custom PCB (described in Section 2) and the 3D printed holder using screws ( Figure 4b).

To maintain proper electrical contact between the antenna and the PCB, it is important to carry out an ultrasonic cleaning procedure for the CPW antenna on the diamond. This process involves a 1-minute immersion in acetone, followed by a 1-minute immersion in ethanol, and finally, a 1-minute immersion in deionized water.

## 4 Results and discussion

**Figure 5.**
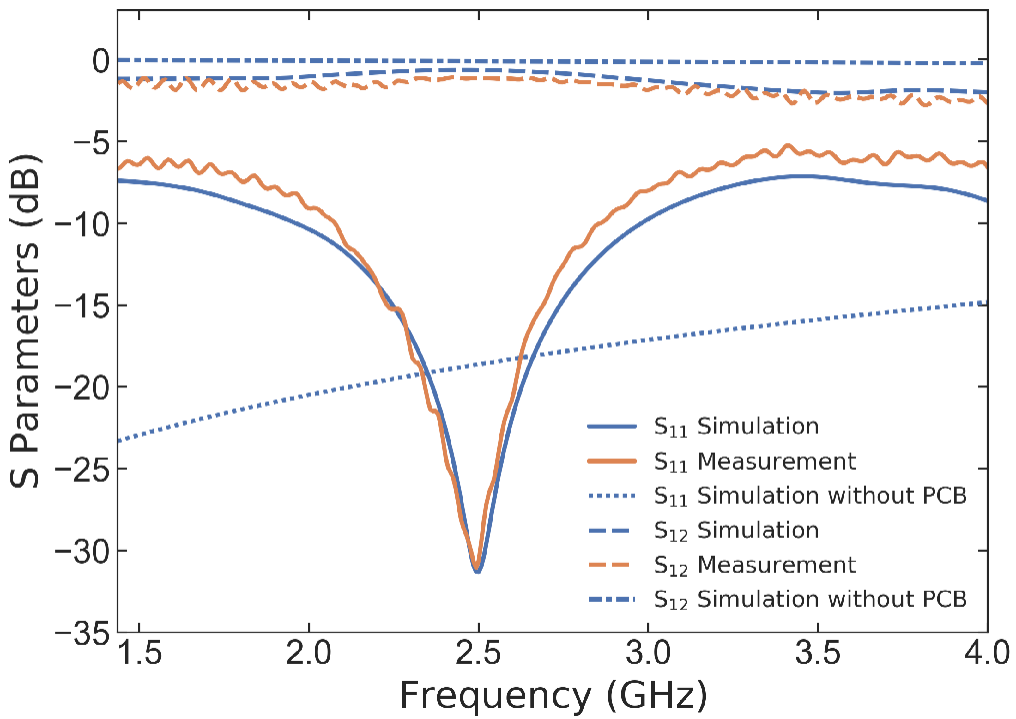
Simulated and measured S-parameters ( *S*_11_ and *S*_12_) of the CPW coil without and with being stacked in between a printed circuit board (PCB) and 3D printed holder. The results demonstrate the high transmission efficiency and low reflection coefficient of the proposed design.

To evaluate the performance of the antenna, we examine the scattering parameters (S-parameters), as shown in Figure 5. The simulated results indicate that when the antenna is excited using an RF probe without a PCB, it can achieve high bandwidth with an *S*_11_ below −10 dB. Moreover, the transmission coefficient is nearly unity, as verified by *S*_21_ ≈ 0 dB. The antenna’s performance in a stack configuration with the PCB and holder has been studied both experimentally and numerically. Figure 5 compares the measurement results with the simulations, demonstrating good agreement between the two. The reflection coefficient results represent a high bandwidth with *S*_11_ *<* −10 dB over a range of 1 GHz, and a resonance frequency at 2.5 GHz is also observed.

**Figure 6.**
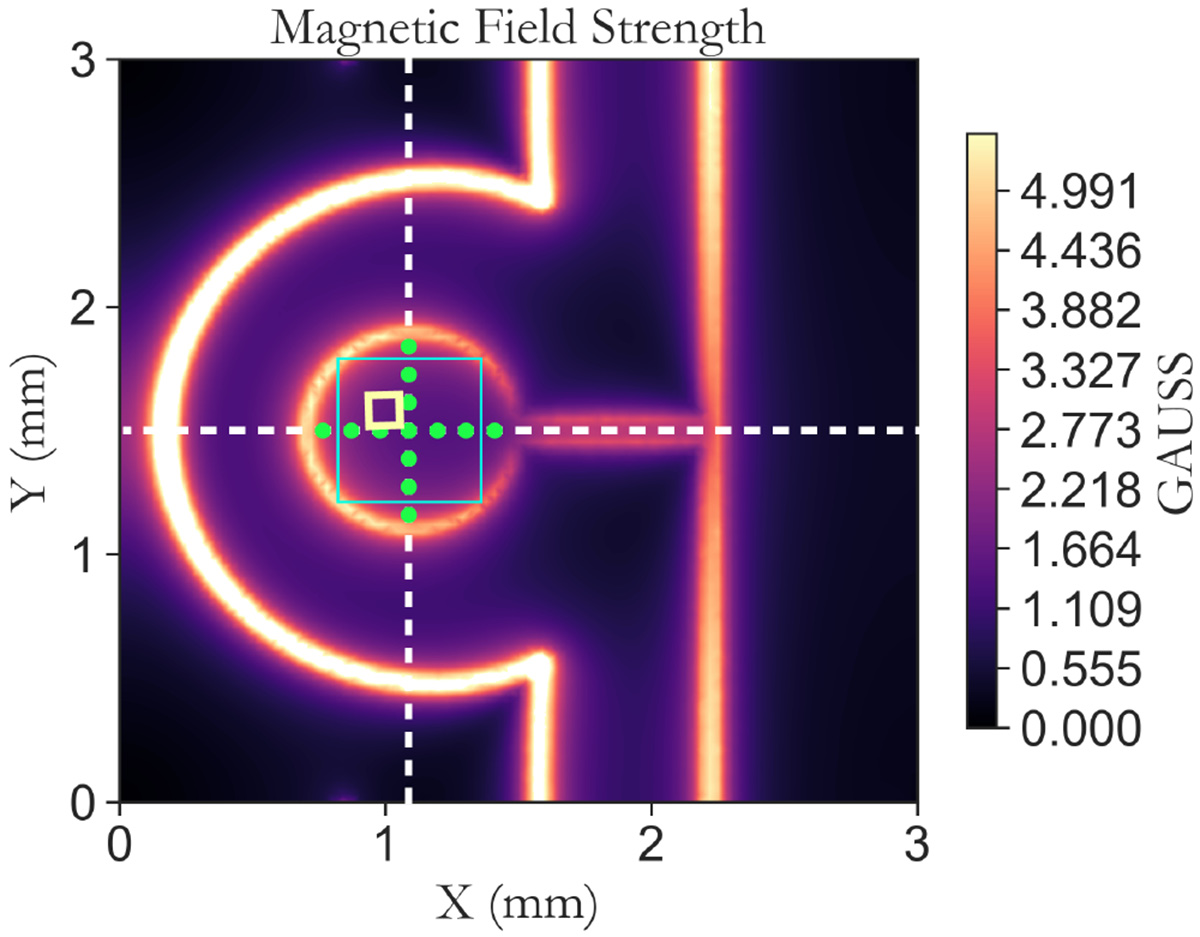
Simulated two-dimensional map of the complex magnitude of the magnetic field (in units of Gauss) at 20 *μ*m inside the diamond for the CPW coil. The dashed white lines represent the cut for plotting x,y,z, and magnitude of the magnetic field in Figure 7. The green circles on the plot represent the optimization points utilized during the simulation and the yellow and cyan squares illustrate the areas to calculate CV and measure Rabi maps, respectively.

The proposed CPW coil is designed to generate a strong and homogeneous *B*_1_ field, which can be evaluated by examining the simulated complex magnitude of the oscillating magnetic field over the diamond sample, as presented in Figure 6. The magnetic field distribution is calculated at a center frequency of 2.87 GHz (corresponding to the NV ^−^ zero-field splitting value in the ground state), on a 2D plane positioned at 20 *μ*m inside the diamond, representing the approximate focal point of the laser used to excite the diamond. In all simulations and measurements, the input microwave power is consistently set at 30 dBm, ensuring a fair comparison across different antenna configurations.

**Figure 7.**
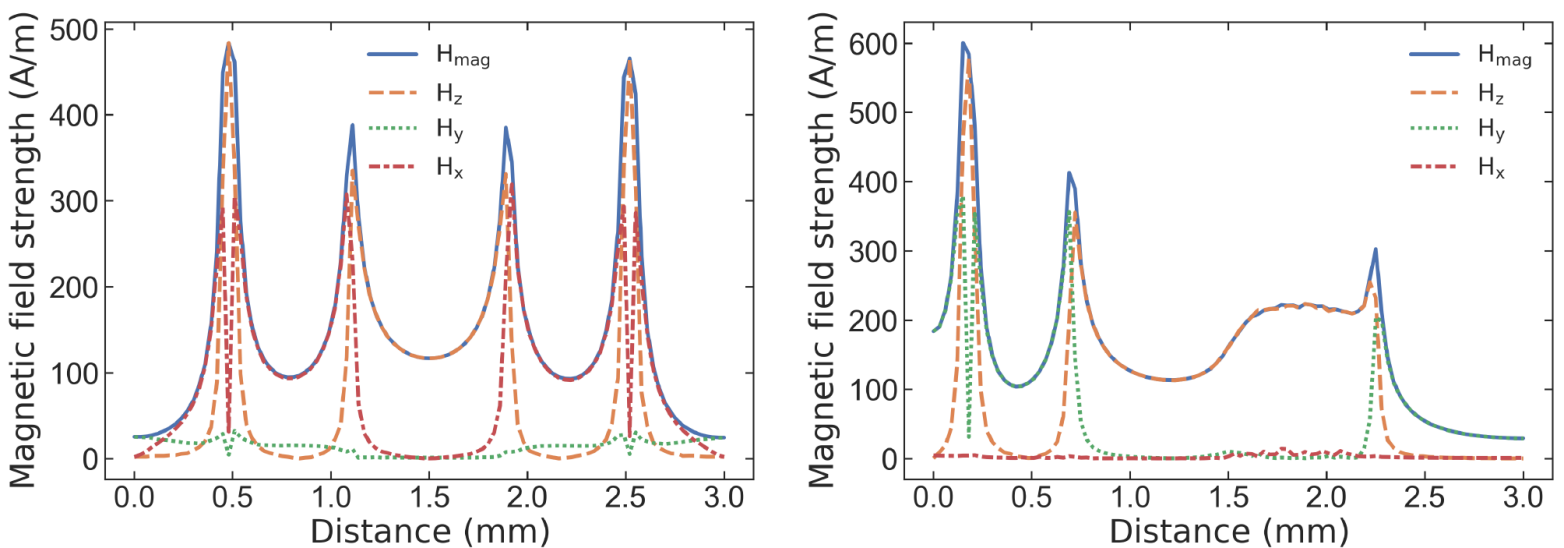
Simulated oscillating magnetic field strength (in units of A/m) along the ( **a**) y-axis and ( **b**) x-axis at 20 *μ*m inside and in the center of the proposed CPW coil . The result is plotted for the x, y, z, and magnitude components of the *B*_1_ field. The results demonstrate the high and homogeneous magnetic field of such a compact structure.

Figure 7 presents the simulated results for the complex magnitude of the oscillating magnetic field and its amplitude along the x, y, and z directions defined in Figure 1a. The result is obtained for the CPW antenna without PCB ( 7a, 7b), to assess the antenna’s capability in generating a high and homogeneous *B*_1_ field. The simulation outcomes reveal that the proposed antenna creates a homogeneous field predominantly oriented along the z-axis (perpendicular to the antenna plane) at the center. This is attributed to the grounding principle of the CPW, where the signal conductor and ground plane reside on the same plane. The magnetic field is generated by the surface current flowing through the planar gold structure, resulting in an increase in the magnetic field at the edges while creating a strong and uniform field in the center. The field is uniform with a coefficient of variation of less than 25% in the whole area (represented by a cyan square in Figure 6a), less than 6% in the area of 0.5mm ^2^, and less than 3.2% in a 100 *μ*m× 100 *μ*m in the center.

**Figure 8.**
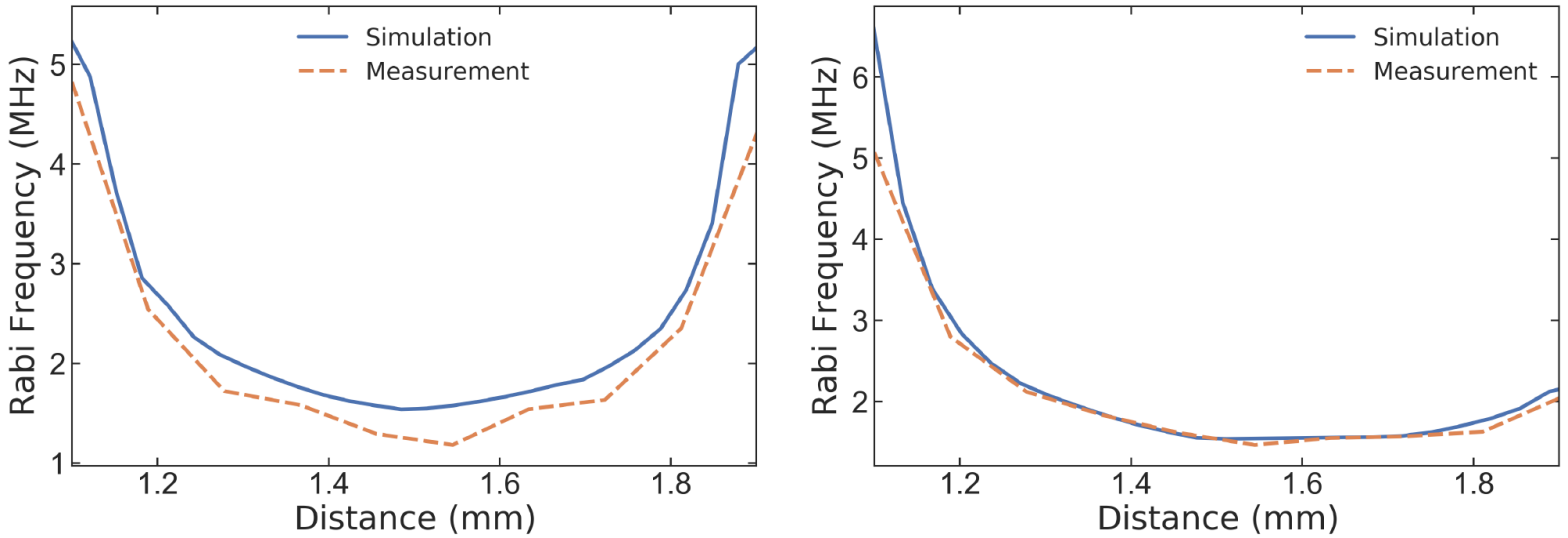
Measured Rabi frequency versus expected simulated Rabi frequency (in units of MHz) along the ( **a**) x-axis and ( **b**) y-axis at 20 *μ*m inside the diamond and in the center of the co-planar waveguide (CPW) microwave coil stacked between PCB and holder.

To validate the simulated results, we perform ODMR and consequently, Rabi measurements using a homemade confocal microscope (see Section 6 for the details of the measurement and methods). The measurements are performed at the center of the planar coil stacked between PCB, 20 *μ*m inside the diamond, along both the x and y axes. The microwave input power is fixed at 1W, and the results are compared to the simulation results, as reported in Figure 8. We observe a great accordance between the experimental and simulated data, with any discrepancies likely attributable to factors such as the assembly of the PCB and CPW. Noteworthy, the Rabi frequency generated by the PCB excitation will be smaller due to impedance mismatching of the feed-line and CPW.

We can determine the relationship between the measured Rabi frequency and the oscillating magnetic field ( *B*_1_) by considering that the rate of transition between *m _s_* = 0 and *m _s_* = ±1 depends on the component of *B*_1_ that is perpendicular to the NV axis (see Section 6 for more details).

**Figure 9.**
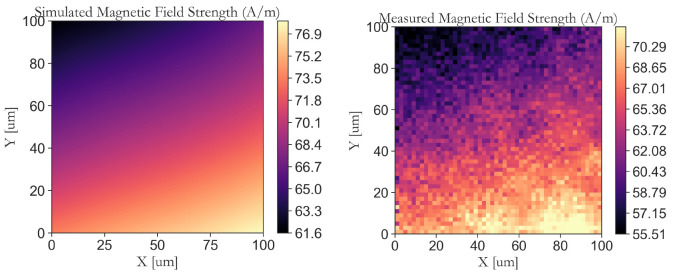
( **a**) Simulated two-dimensional map of the magnetic field (in units of Ampers per meter) at a distance of 30 *μ*m off from the center of the proposed CPW coil (Map is obtained within the yellow square as depicted in Figure 6a). The map shows the spatial distribution of the oscillating magnetic field in the vicinity of the CPW, which is critical for efficient spin manipulation of diamond nitrogen-vacancy (NV) centers. ( **b**) Measured two-dimensional map of the magnetic field (in units of Ampers per meter) at a distance of 30 *μ*m off from the center of the proposed CPW coil. The measurement is performed by measuring Rabi frequency at each position on NV centers that have an angle of 55.81° relative to the applied DC magnetic field.

The simulated and measured magnetic field maps at an offset distance of 30 *μ*m from the center of the CPW coil are shown in Figure 9a and Figure 9b, respectively. These maps are conducted at 20 *μm* inside the diamond and reveal the spatial distribution and homogeneity of the generated magnetic field by the planar CPW coil. Calculating the coefficients of variations for maps in Figure 9a we demonstrate a notably low value of 5.4% and 6.5% for the simulation and measurement, respectively. To obtain these measurements, a piezo stage from Mad City Labs is used to move the sample across a 100 *μ*m× 100 *μ*m area. At each point, the Rabi frequency is measured, and the data is then converted to the corresponding magnetic field in units of A/m (see Section 6). This process has enabled a direct comparison between the measured magnetic field maps and the simulated maps.

## 5 Conclusion

In conclusion, this paper has presented a novel CPW gold coil patterned on a 3 × 3 mm ^2^ CVD diamond substrate to address the limitations and challenges associated with earlier antenna designs for NV ensemble magnetometry applications. The proposed CPW coil offers a highly homogeneous magnetic field over a large area of 0.5 mm ^2^, full integration with a diamond NV center sensor, broad bandwidth from 1.5 to 4 GHz, and ease of testing and operation through the proposed PCB and holder designs. The optimized critical parameters of the coil design, such as the omega-shaped resonator diameter, waveguide width, and gap size, ensure the desired homogeneous magnetic field. Experimental results, including Rabi measurements and S-Parameter analysis, have shown good agreement with simulation results, validating the performance of the proposed CPW coil.

This research has detailed the antenna design and geometry, fabrication process, construction of the PCB stack, and experimental evaluation of the antenna’s performance. The proposed CPW coil holds significant potential for advancing the field of NV magnetometry, combining homogeneity, broad bandwidth, and ease of use with the possibility of full integration on a diamond substrate. Future studies may explore additional optimizations or adaptations to further enhance the performance and applicability of the CPW coil in ensemble NV magnetometry applications combined with optimized collection and excitation efficiency.

## 6 Methods

**Figure 10.**
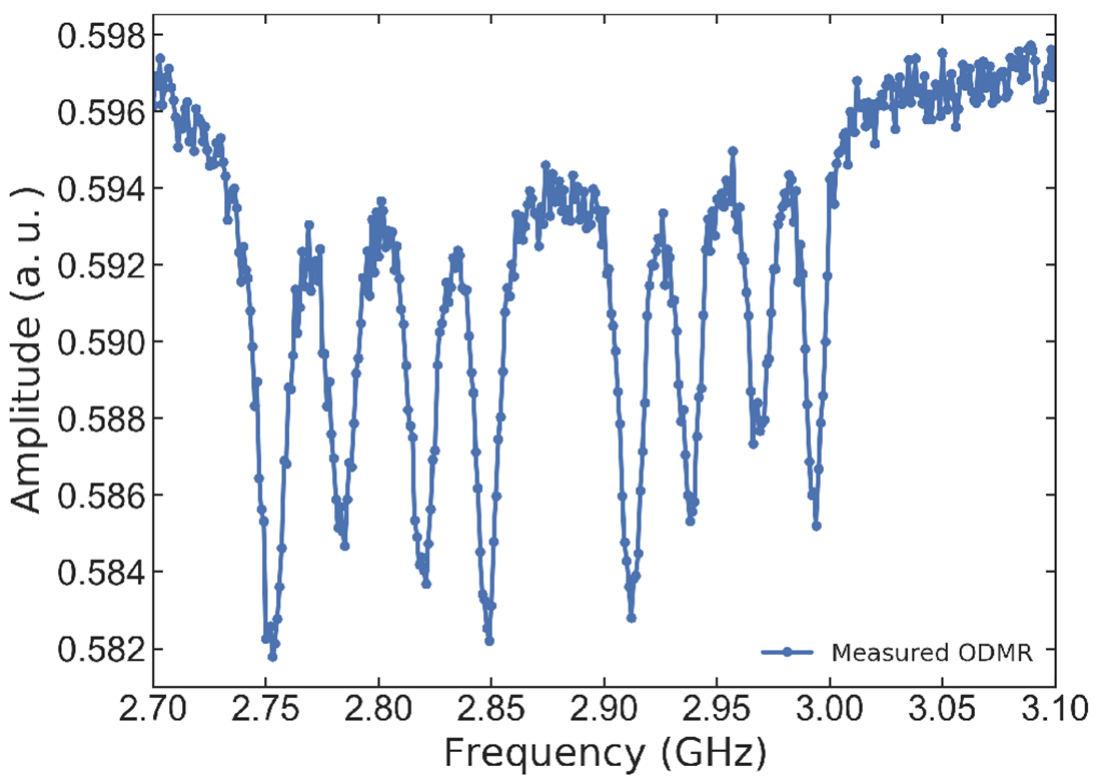
Optically detected magnetic resonance (ODMR) obtained applying the MW field using our antenna and PCB.

CW-ODMR measurements are performed to explore the application of our antenna in controlling ensembles of NV centers in diamond. These measurements are carried out in a homemade confocal microscope, more details about this set-up can be found in 19. To this, a biased magnetic field using a permanent magnet is applied to achieve non-degenerately splitting for all NV axes. The NV centers are excited using a 515 nm laser (Cobalt-06-MLD), and the resultant fluorescence signal is captured with an avalanche photodiode (Thorlabs APD430A). Concurrently, a microwave signal sweep calibrated at 1W is applied to the printed circuit board of the CPW coil using a Rohde and Schwarz SMF100A microwave signal generator. The resulting signal is synchronously captured by a National Instruments data acquisition card (NI 6361) during photon detection. When the microwave frequency matches the spin transition, the NV center undergoes a spin flip, resulting in a decrease in the fluorescence intensity. Figure 10 displays the ODMR signal, which features eight dips corresponding to the four possible orientations of the NV centers within the diamond lattice. The observed ODMR signal can be interpreted using the Hamiltonian approximation given below ^1, 15^:

$$H=D\left(S_{z}^{2}\right)+\gamma_{NV}B_{0}\text{(}\sin\text{(}\alpha \text{)}S_{x}+\cos\text{(}\alpha \text{)}S_{z}\text{)}\;\text{(}1\text{)}$$

In Equation 1, $\gamma_{\text{NV}}=\frac{\mu_{B}g_{S}}{h}$ = 2.8 MHz/Gauss represents the gyromagnetic ratio, *S* is the spin matrix, and *α* is the angle between the NV center axis and the applied DC biased magnetic field, *B*_0_. By analyzing the ODMR signal and solving the Hamiltonian for all four NV axes, it is possible to numerically determine the orientation and magnitude of the applied magnetic field.

The numerical solution script for finding *B*_0_ and its angle relative to all NV axes is provided at the following link: . Our results indicate that for the ODMR presented in Figure 10, the DC magnetic field has a value of 77.41 Gauss. The angles corresponding to the ODMR dip pairs, ordered from the highest to the lowest splitting value, are 55.81°, 115.64°, 106.09°, and 82.45°, respectively.

**Figure 11.**
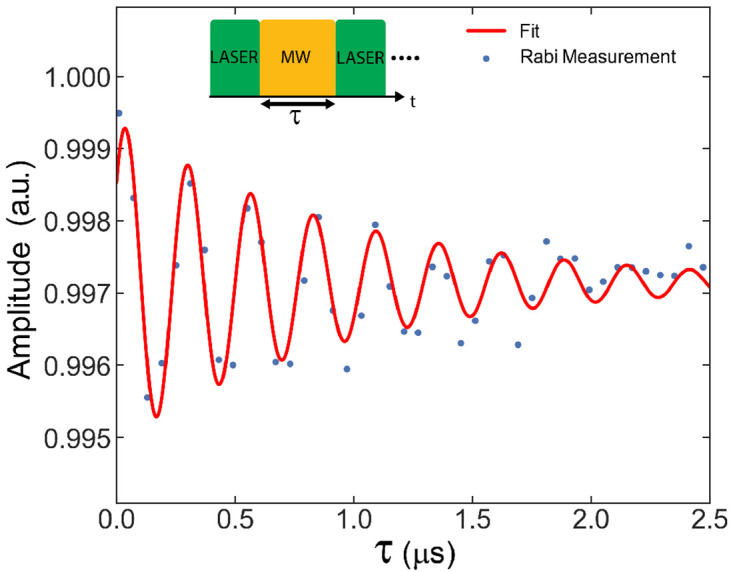
Rabi oscillation measurement for the NV axis at an angle of 55.81° relative to the DC magnetic field within the CPW microwave coil. The Rabi period and lifetime are estimated from the fit as 264 ns and 0.9 *μ*s respectively.

To evaluate the coupling efficiency of the microwave field generated by the antenna to the NV centers in diamond, we carried out a Rabi measurement. Rabi measurement describes the coherent oscillation between the energy levels of the NV center and is performed within a pulse measurement experiment. To this, laser pulses with a duration of 100 *μ*s along with applied microwave pulses with a calibrated input power of 1 W to the antenna port are utilized. The time span between two consecutive laser pulses is occupied by a microwave pulse that is in resonance with the chosen NV transition. The duration of the microwave pulses varies linearly. During the period when the laser pulse is applied, the photoluminescence (PL) is averaged out to yield a more accurate readout. The measurements are synchronized using a Swabian pulse streamer instrument. A triggered readout is achieved with the assistance of an NI data acquisition card. By identifying the relevant captured pulse associated with alterations in the distance between laser pulses, we can determine the relative occupation of the spin state transitioning from *m _s_* = 0 to *m _s_* = ±1. This can be found by calculating the ratio between the end of the detected pulse and the beginning of the pulse. The ratio for each time delay, with a range spanning from 0 to 2.5 *μ*s and a spin transition frequency of 2.848 GHz, is depicted in Figure 11. Figure exhibits a Rabi oscillation of 3.9 MHz at the focal plane of the objective, situated 20 *μ*m within the sample. The measured Rabi frequency is fitted with a damped cosine function, as expressed by Equation 2.

$$F\left(t\right)=A\text{cos}\text{(}2\pi f_{R}t+\varphi_{0})e^{-\zeta_{D}t}+B\;\text{(}2\text{)}$$

where *f _R_* is Rabi frequency and *ζ _D_* is the decay rate of the Rabi oscillation.

The Rabi frequency can also be expressed as *f _r_* = $\frac{1}{\sqrt{2}}$ *γB*_1_ sin *β*, where $\frac{1}{\sqrt{2}}$ accounts for the rotating wave approximation in a spin 1 system and *β* is the angle between *B*_1_ and the corresponding NV axis ^16^. If the CW-ODMR splitting and *B*_1_ direction from a simulation are known, it is possible to determine *B*_1_ from the Rabi frequency.

## Data Availability

Source code available from: https://github.com/hbabashah/hbabashah.github.io. Archived source code at time of publication: https://doi.org/10.5281/zenodo.10401685 ^20^. License: MIT License.
