# Supplementary material for: Integrated coplanar waveguide coil on diamond for enhanced homogeneous broadband NV magnetometry
Source: Open Res Eur. 2024 Mar 1;4:44. [Version 1] doi: 10.12688/openreseurope.16875.1 (PMC11325137; doi:10.12688/openreseurope.16875.1)
Supplement: Peer review history

# Reviewer report: Reviewer response for version 1

**DOI:** 10.21956/openreseurope.18234.r39818

**Published:** 2024-08-16

**Authors:** Bing Chen

**Affiliations:** 1 Hefei University of Technology, Hefei,, Anhui, China

The paper presents a coplanar waveguide (CPW) gold coil patterned on a 3 × 3 mm2 diamond substrate, offering full integration, enhanced stability, and broad bandwidth suitable for various NV sensing applications. The authors include more comprehensive details about the CPW on diamond.

 1. What is the difference between CPW and double split-ring antenna.

 2. Will plating electrodes directly on the diamond cause the metal to penetrate into the diamond?

 3.Why is the resonant point of this coplanar waveguide not 2.87GHz.

Is the study design appropriate and does the work have academic merit?

Yes

Is the work clearly and accurately presented and does it cite the current literature?

Yes

If applicable, is the statistical analysis and its interpretation appropriate?

Yes

Are all the source data underlying the results available to ensure full reproducibility?

Yes

Are the conclusions drawn adequately supported by the results?

Yes

Are sufficient details of methods and analysis provided to allow replication by others?

Yes

Reviewer Expertise:

quantum sensing using NV center in diamond

I confirm that I have read this submission and believe that I have an appropriate level of expertise to confirm that it is of an acceptable scientific standard.

# Reviewer report: Reviewer response for version 1

**DOI:** 10.21956/openreseurope.18234.r39824

**Published:** 2024-08-14

**Authors:** Suvechhya Lamichhane

**Affiliations:** 1 University of Nebraska Lincoln, Lincoln,, Nebraska, USA

This study has effectively implemented a coplanar waveguide (CPW) directly onto the diamond substrate, showcasing a uniform field distribution across a wide area. This uniform microwave field significantly enhances sensitivities for ensemble NV magnetometry, rendering the work noteworthy in the NV sensing community. The experiments and simulations are well conducted to demonstrate the efficacy of their design. However, certain aspects require clarification for result reproducibility and interpretation.

1. The reason behind utilizing Ti atop the Au layer remains unclear. Additionally, specifics regarding the electron beam's acceleration voltage and exposure dose are indispensable. Are there any concerns regarding charge accumulation during e-beam exposure?
2. Discrepancies between the simulated magnetic field strength (~1.6 Gauss in Figure 6) and the field estimation from Rabi data (~0.95 Gauss in Figure 9) necessitate clarification. It would be nice to have the explanation for this inconsistency.

Is the study design appropriate and does the work have academic merit?

Yes

Is the work clearly and accurately presented and does it cite the current literature?

Yes

If applicable, is the statistical analysis and its interpretation appropriate?

Not applicable

Are all the source data underlying the results available to ensure full reproducibility?

Yes

Are the conclusions drawn adequately supported by the results?

Partly

Are sufficient details of methods and analysis provided to allow replication by others?

Partly

Reviewer Expertise:

NV magnetic sensing

I confirm that I have read this submission and believe that I have an appropriate level of expertise to confirm that it is of an acceptable scientific standard, however I have significant reservations, as outlined above.

# Reviewer report: Reviewer response for version 1

**DOI:** 10.21956/openreseurope.18234.r39504

**Published:** 2024-04-23

**Authors:** Jixing Zhang

**Affiliations:** 1 University of Stuttgart, Stuttgart, Germany

This research article introduced an integrated coplanar waveguide coil on diamond. The study meticulously details the design, fabrication process, and experimental evaluation of the proposed antenna, showcasing its potential for enhancing magnetic field homogeneity and broad bandwidth in NV magnetometry applications. The authors' thorough analysis, supported by simulation and experimental results, demonstrates the effectiveness of the CPW coil in achieving desired performance metrics.

 While the design of CPW and its application for NV centre in diamond is a well-explored field, this paper lacks significant innovation in CPW design. However, it still holds value for society by providing a detailed fabrication process. Therefore, I recommend that the authors include more comprehensive details about the fabrication of CPW on diamond to ensure reproducibility by others.

 Moreover, the direct printing of CPW on diamond offers improved heat dissipation but also raises concerns about diamond heating. Given that the zero field splitting of NV centres is sensitive to temperature fluctuations, it would be beneficial to discuss this effect further.

Is the study design appropriate and does the work have academic merit?

Partly

Is the work clearly and accurately presented and does it cite the current literature?

Yes

If applicable, is the statistical analysis and its interpretation appropriate?

Yes

Are all the source data underlying the results available to ensure full reproducibility?

Partly

Are the conclusions drawn adequately supported by the results?

Yes

Are sufficient details of methods and analysis provided to allow replication by others?

Yes

Reviewer Expertise:

nitrogen vacancy center based quantum sensing

I confirm that I have read this submission and believe that I have an appropriate level of expertise to confirm that it is of an acceptable scientific standard, however I have significant reservations, as outlined above.

# Reviewer report: Reviewer response for version 1

**DOI:** 10.21956/openreseurope.18234.r38631

**Published:** 2024-04-22

**Authors:** Wojciech Gawlik

**Affiliations:** 1 Jagiellonian University, Krakow, Poland

The article by H.Babashah, E. Loser, and Ch. Galland describes the design of a waveguide coil for NV magnetometry on the surface of a bulk diamond crystal. In addition to the discussion of the main design criteria, the authors verified their predictions in the experiment and discussed the results obtained.

 The creation of a homogenous, intense, and broadband microwave field is a crucial issue for practical magnetometry with NV diamonds, so there is a need for successful designs that meet as much as possible of the above criteria. The described construction largely satisfies these requirements. It produces a reasonably strong field in a broad frequency band. Although the area of homogeneity is not very impressive, the important advantage is that the constructed coil is placed directly on the diamond crystal, so it is fully integrated with a diamond crystal, which is very advantageous from the point of view of cooling and compactness of the device.

 Discussion of the system performance based on S-parameter analysis of the S parameters is very detailed and accurate. I was pleased with the careful elimination of the higher-order mode by a careful and well-thought-out discussion of the coil dimensions. Figures 7-11 illustrate that the constructed coil meets expectations, so it can be recommended as a useful and convenient design. 

 When reading the paper, I noticed some problems referring to Figure 6a in Sect.2, page 4. Apparently, Figure 6a does not exist. Perhaps the authors meant Figure 7, when they referred to ‘green circles’, but I only found green dots there, rather than green circles. Also, there were no labels (a), (b) associated with two figures 7, so this part of the text required some effort to follow. 

 In conclusion, the article discusses criteria important for the construction of an NV magnetic sensor and presents a successful design that can be recommended for applications by other researchers.

Is the study design appropriate and does the work have academic merit?

Yes

Is the work clearly and accurately presented and does it cite the current literature?

Yes

If applicable, is the statistical analysis and its interpretation appropriate?

Yes

Are all the source data underlying the results available to ensure full reproducibility?

Yes

Are the conclusions drawn adequately supported by the results?

Yes

Are sufficient details of methods and analysis provided to allow replication by others?

Yes

Reviewer Expertise:

Atomic and laser physics, spectroscopy research on warm and cold atoms and colour centers in diamonds

I confirm that I have read this submission and believe that I have an appropriate level of expertise to confirm that it is of an acceptable scientific standard.
